# The difference of stress distribution of maxillary expansion using rapid maxillary expander (RME) and maxillary skeletal expander (MSE)—a finite element analysis

**DOI:** 10.1186/s40510-018-0229-x

**Published:** 2018-10-03

**Authors:** Nathania Hartono, Benny M. Soegiharto, Retno Widayati

**Affiliations:** Department of Orthodontics, Faculty of Dentistry, University of Indonesia, Jl, Salemba Raya no. 4, Jakarta Pusat, 10430 Indonesia

**Keywords:** Finite element analysis (FEA), Rapid palatal expander (RME), Maxillary skeletal expander (MSE), Stress distribution

## Abstract

**Background:**

Maxillary skeletal expander (MSE) in combination with miniscrews was developed to overcome the drawbacks that may have resulted from the application of conventional rapid maxillary expander (RME). This research was conducted to analyze the difference of stress distribution of maxillary expansion using RME and MSE in the region of interests (ROIs): first molars (M1), palatal alveolar bones of M1, palatine sutures, zygomatic sutures, miniscrews, and their surrounding bones.

**Methods:**

A dry skull was scanned using CBCT and rendered into a three-dimensional (3D) model of craniomaxillary structures. The data analysis was done both visually and numerically.

**Result:**

The stress distributions in RME group were located at the palatal side of M1, mesial side of palatal alveolar of M1, pulp chamber of M1, and inferior cortex of palatine sutures. The stress distributions in the MSE group were located at the distopalatal cusp of M1, palatal side of palatal alveolar of M1, and inferior and superior cortex of palatine sutures. The stress distributions in zygomatic sutures on both groups were located at the zygomaticotemporal sutures, whereas in the miniscrews, the stress were located at the anterior miniscrews and palatal side of surrounding bones.

**Conclusions:**

There were significant differences of stress distribution of maxillary expansion measured in the ROIs in the craniomaxillary 3D model using RME and MSE.

## Background

Maxillary expansion is a common procedure for the management of patients with transverse maxillary deficiency which was a challenging problem for clinicians. The first expansion method was described by E.C. Angell in 1860 and was later developed by T.M. Graber in 1940 to treat cleft lip and palate patients. This expander was then popularized by Korkhaus and Andrew Haas and becomes the treatment of choice for patients with constricted maxillary arch [1].

The aim of maxillary expansion was to optimize the dentofacial orthopedics effect while minimizing the dentoalveolar side effects, thus the total expansion obtained through the usage of rapid maxillary expansion, can be categorized into skeletal expansion, alveolar expansion, and bending or tipping of the teeth [2, 3].

Previous studies described a reduction on the cortical bone of posterior teeth after the use of rapid maxillary expander. The reduction of cortical bone was even more pronounced on the teeth that served as direct anchorage to the expander, which was attributed to the tipping movement of the teeth [3–5]. However, this comes with several disadvantages, such as age limitation, dentoalveolar tipping, root resorption, and bone dehiscence, as well as, the lack of long-term stability [6]. Wehrbein et al (1996) firstly introduce the used of miniscrews in palatal area because palatal was covered with keratinized gingiva and gave good flexibility [7–9]. Weissheimer (2011) reported that the used of RME alone gave smaller effects at the skeletal level and Lagravere et al (2010) reported the effects of rapid maxillary expander with bone anchorage such as there was less dental tipping than the usage of rapid maxillary expander alone. Those studies reported that the usage of miniscrew-assisted rapid maxillary expander were effective in preventing the negative side effects that were commonly seen with the usage of rapid maxillary expander alone [10, 11]. Therefore, many clinicians opted miniscrews as non-invasive expansion alternative method.

With the innovation of miniscrews, it is now possible to reinforce the anchorage system of rapid maxillary expander without the support of tooth structure because miniscrews serve as the orthodontic absolute anchorage. Bone anchored rapid maxillary expander were reported to transmit a direct expansion force to the palatal bone, which contribute in a more skeletal opening of the suture, instead of bending of the maxillary alveolar bone as the force vector located near the bone [2, 12]. Lagravere et al (2010) concluded no significant difference was found between bone-borne and tooth-borne rapid maxillary expander. A study conducted by Lee at al in 2014 using a bone-borne expander with miniscrew, showed a different characterictic [13]. Mosleh et al (2015) stated that the expansion force on palatal bone will produce a more parallel opening, without causing unwanted tooth movement [14]. 

Maxillary skeletal expander (MSE) is manufactured by Biomaterial Korea Inc. It comprises of two molar bands and body that include an expansion screw with four welded tubes. Each of the tube was 1.5 mm in diameter, and 2 mm in length, that facilitates the placement of the miniscrew. The miniscrew is 1.5 mm in diameter and 11 mm in length. The tube and the miniscrew had the same diameter to minimize lateral forces that might affect the molar teeth [15]. 

Lin (2015) conducted a study that compares tooth-borne and bone-borne rapid maxillary expanders in late adolescence using CBCT, reported both expanders produced expansion, but maxillary skeletal expander produced greater orthopedic effects and a more parallel opening of the suture. Subjects in MSE group showed less change of alveolar inclination and tooth axis compared to subjects in RME group. The change of teeth angulation was a combination of bone bending and tipping of the teeth. As teeth were surrounded by alveolar bone and undergoing remodeling process, it was hard to objectively separate bone bending and tipping of the teeth. The lesser tipping found in MSE can be explained with the use of skeletal anchorage. The 11 mm length miniscrews used in MSE increase the stability of the expander as the miniscrew engage both cortical bone in the oral and nasal floor [15].

Various research of miniscrew-assisted maxillary expansion have been conducted using photo elastic model analysis, holography laser, and computed tomography or Cone-Beam Computed Tomography (CBCT) [2, 11, 13, 16, 17]. The results showed significant maxillary expansion indicated by significant increase in interpremolar and intermolar width. Moreover, a more parallel opening of the palatine suture was found, compared to those found with the usage of rapid maxillary expander alone [16]. Previous research showed that both rapid maxillary expander and miniscrew-assisted rapid maxillary expander resulted in dental and skeletal changes.

In orthodontics biomechanics, aside from macro force system, micro mechanical data such as dental stress distribution, periodontal ligament, and alveolar bone are indispensable factors to understand biological property of tooth movement, root resorption, and bone remodeling. Structure and biological property of oral cavity are highly complex, thus analytic methodology could not resolve micro mechanics problems. Computer simulation using finite element analysis (FEA) could adapt complex structure, making FEA popular in biomechanics field, including orthodontic biomechanics. Over the last few years, FEA become a significant method to understand tooth and tissue response to force at a biomechanical level [18, 19]. In order to accurately perform such simulations, a detailed understanding of material property of each structures tested is highly important. Material property hugely affects stress distribution and strain within structure. Isotropic materials are defined by two constants, e.g. *Young’s modulus* and *Poisson’s ratio* [20] (Table 1). This study compares the stress distribution of the upper first molar, palatal bone of upper first molar, palatine suture, and zygomatic suture with conventional rapid maxillary expander and miniscrew-assisted maxillary skeletal expander using finite element analysis.

**Table 1:** Young’s modulus and Poisson’s ratio of various materials used in this study

No	Material	Young’s modulus	Poisson’s ratio
1	Maxilla skeletal expander (MSE)	193.000 MPa	0.3
2	Rapid maxillary expander (RME)	200.000 GPa	0.33
3	Miniscrew (Ti-6Al-4V: titanium 6% alumunium 4% vanadium)	114.000 MPa	0.34
4	Alveolar bone (cortical bone)	13.700 MPa	0.3
5	Cancellous bone	1.370 MPa	0.3
6	Suture	0.068 MPa	0.49
7	Teeth	20.700 MPa	0.30
8	Enamel	80.000 MPa	0.25
9	Dentine	18.300 MPa	0.30
10	Pulp	0.58 MPa	0.42
11	Periodontal ligament	0.7 MPa	0.49

All materials in the model were assumed to be homogenous, isotropic, and linearly elastic

## Methods

In this study, a dried human skull was obtained from the Faculty of Medicine and was scanned using CBCT machine (Orthophos SL 3D; Sirona, German) with 0.4-mm voxel size and 22-mm field of view (FOV). A three-dimensional model of craniomaxilla was then created by assembling the tooth elements, maxillary alveolar bone, palatal bone, and cranial bone. The object was stress distribution to the upper first molar, palatal bone of upper first molar, palatine suture, zygomatic suture, miniscrews, and palatal bone around inserted miniscrews when expansion force was applied through a jackscrew. Population of the research was all nodes found on 3D craniomaxillary model, and the samples were nodes found on the first upper molar, palatal alveolar bone of first upper molar, palatine suture, zygomatic suture, miniscrews, and palatal bone around inserted miniscrews. The material for this research was a set of dried human skull, a set of maxillary skeletal expanders, four pieces of miniscrews of 1.5 mm diameter and 11 mm long, and a set of rapid maxillary expander.

Ethical approval was granted by the ethics committee at the Faculty of Dentistry University of Indonesia. Palatal bone, skull, maxillary alveolar bone, and each dental element were assembled to create a solid 3D craniomaxillary model. The maxillary skeletal expander, rapid maxillary expander, and miniscrews were created in conformity with the originals. Unigraphics software was used to create all the tools. Mesh was automatically generated using ANSYS software (17.1, ANSYS Inc.). To determine the expansion effect on the stress distribution, several openings of the jackscrew were investigated. FEA was to measure stress distribution on the upper first molar, palatal bone of upper first molar, palatine suture, zygomatic suture, miniscrews, and palatal bone around inserted miniscrews while several expansion forces are being applied. ANSYS software was used to visually determine the stress distribution when expansion force are being applied. The expansion force investigated in this study are two turns of jackscrew rotation, four turns of jackscrew rotation, and six turns of jackscrew rotation which created 0.5, 1, and 1.5 mm, respectively, of displacement. Simulation was conducted three times for each model to determine MaxPs (highest tension), MinPs (highest compression), and von Mises.

## Results

In this study, the data was analyzed both visually and numerically on each region of interest (ROI), which were on the upper first molar, palatal bone of maxillary first molar, palatine suture, zygomatic suture, miniscrews, and palatal bone around miniscrews insertion site, where a total of 20,250 nodes were tested. Visually, the stress distribution and concentration are employed along with a color map, where red shows the greatest stress concentration and blue shows the least stress concentration. The means and standard deviations of the MaxPS, MinPS, and von Mises of each model were calculated. Statistical significance was determined at the *p* < 0.05 level. The statistical analysis was performed using SPSS software, version 21 (SPSS Inc., Chicago, IL, USA).

**Fig. 1:**
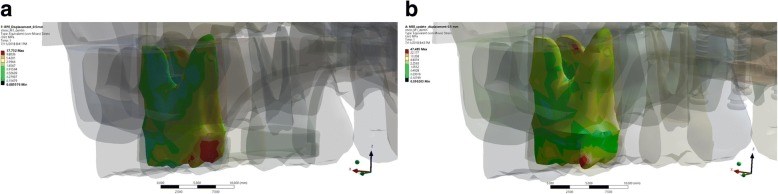
The von Mises stress distribution on the maxillary first molar in the expansion simulation using (**a**) RME, and (**b**) MSE

Visually, there was a difference in the location of stress concentration of upper first molar in RME group and MSE group. The application of expansion force in RME group displayed a stress concentration localized at the enamel and dentine of the palatal part of the upper first molar. On the other hand, the stress concentration was found in the enamel and dentine of distopalatal cusp of upper first molar in the MSE group (Fig. 1).

Normal distribution was taken for all data, and all data were normal. Independent *t* test was performed to compare each type of stress, i.e., von Mises stress, MaxPS, and MinPS generated on various displacements between two groups. The statistical analysis showed a significance value of 0.00 (*p* < 0.05). Therefore, a statistically significant difference was found between groups (Table 2).

**Table 2:** Independent *t* test of the nodes on various displacement of expansion using RME and MSE (region of interest: upper first molar)

Expansion method	Stress	Displacement	*N*	Mean	SD	CI 95%		*p*
						Lower	Upper	
RME	Von Ms	0.5 mm	225	10.86904	1.181594	1.03669	1.38926	0.00^*^
MSE			225	9.65606	0.641251			
RME	Von Ms	1 mm	225	1.49844	0.015366	− 5.23739	− 5.21068	0.00^*^
MSE			225	6.72249	0.100525			
RME	Von Ms	1.5 mm	225	5.74535	0.128029	0.04932	0.0872	0.00^*^
MSE			225	5.67708	0.067008			
RME	MaxPs	0.5 mm	225	4.31042	0.257529	− 4.54820	− 4.39736	0.00^*^
MSE			225	8.78321	0.514199			
RME	MaxPs	1 mm	225	1.80901	0.044066	− 0.76577	− 0.75060	0.03^*^
MSE			225	2.56719	0.037552			
RME	MaxPS	1.5 mm	225	11.36281	0.592480	− 15.3542	− 14.6147	0.00^*^
MSE			225	26.34729	2.752635			
RME	MinPS	0.5 mm	225	− 0.09651	0.012786	0.14908	0.15695	0.00^*^
MSE			225	− 0.24953	0.027169			
RME	MinPS	1 mm	225	− 0.72524	0.014304	1.00171	1.01227	0.00^*^
MSE			225	− 1.73224	0.037582			
RME	MinPS	1.5 mm	225	− 0.45977	0.029023	0.19041	0.2124	0.00^*^
MSE			225	− 0.66117	0.078496			

Independent *t* test, statistical significance was determined at the *p* < 0.05 level

*Significant at *p* < 0.05 level

**Fig. 2:**
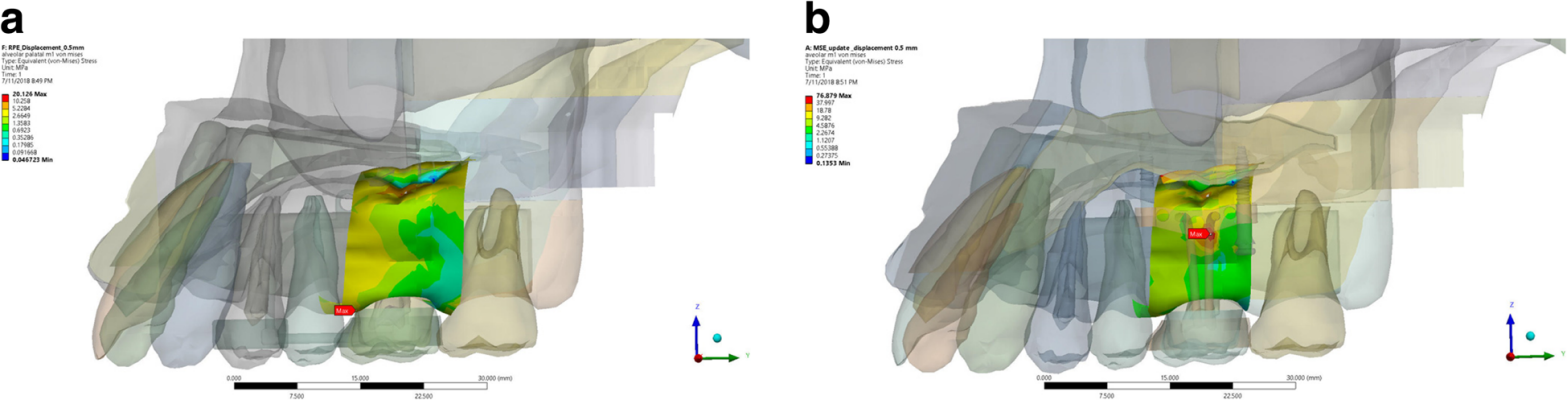
The von Mises stress distribution on the palatal alveolar bone of upper first molar in the expansion simulation using (**a**) RME, and (**b**) MSE

The expansion simulation using RME and MSE showed a similar stress distribution on the palatal alveolar bone of the upper first molar. Von Mises stress distribution showed the highest stress on the mesial alveolar bone of the upper first molar in the RME group, while in the MSE group, the highest stress was found on the palatal alveolar bone of the upper first molar. MaxPS distribution showed the highest tension on the mesial alveolar bone of the upper first molar in the RME group, while in the MSE group, the highest tension was found on the apical part of the palatal alveolar bone of the upper first molar. MinPS distribution showed the highest compression on the mesial alveolar bone of the upper first molar in the RME group, while in the MSE group, the highest compression was found on the bifurcation of the palatal alveolar bone of the upper first molar. The statistical analysis showed a significance value of 0.00 (*p* < 0.05). Therefore, a statistically significant difference was found in each group (Fig. 2).

**Fig. 3:**
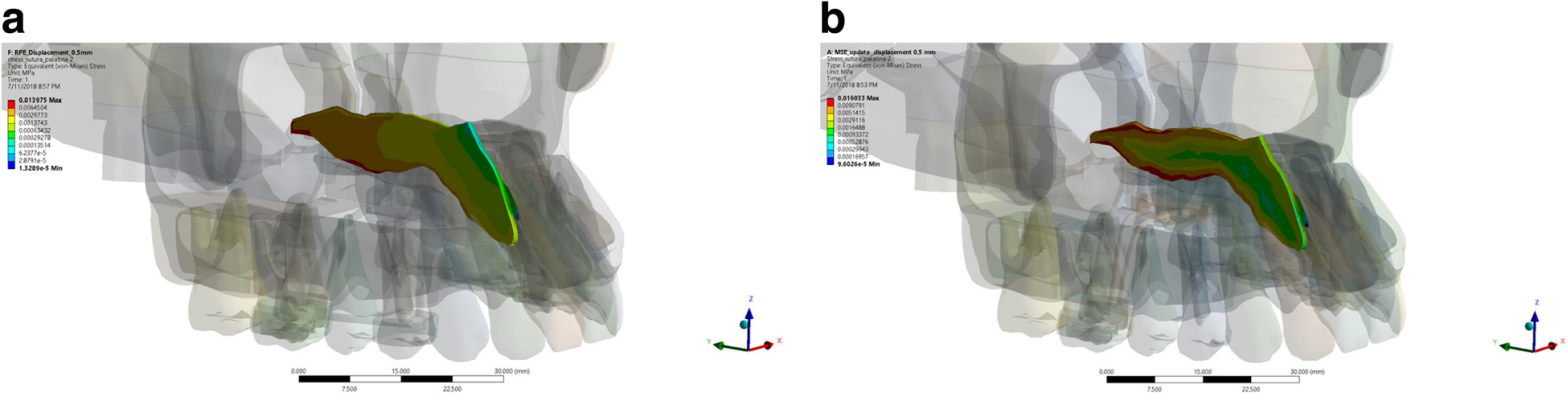
The von Mises stress distribution of the palatine suture in the expansion simulation using (**a**) RME, and (**b**) MSE

Visually, there was a difference in the location of stress concentration of the palatine suture in the RME group and MSE group. The application of expansion force in the RME group displayed a stress concentration localized at the inferior cortex of the palatine suture. In contrast, the stress concentration was found scattering at the inferior and superior cortex of the palatine suture in the MSE group. MaxPS distribution showed the highest tension at the posterior end of palatine suture in the RME group and at the superior cortex of palatine suture in the MSE group. MinPS distribution observed in the palatine suture showed the highest compression is located at the superior cortex in the RME group, in contrast to that at the inferior cortex observed in the MSE group (Fig. 3).

**Fig. 4:**
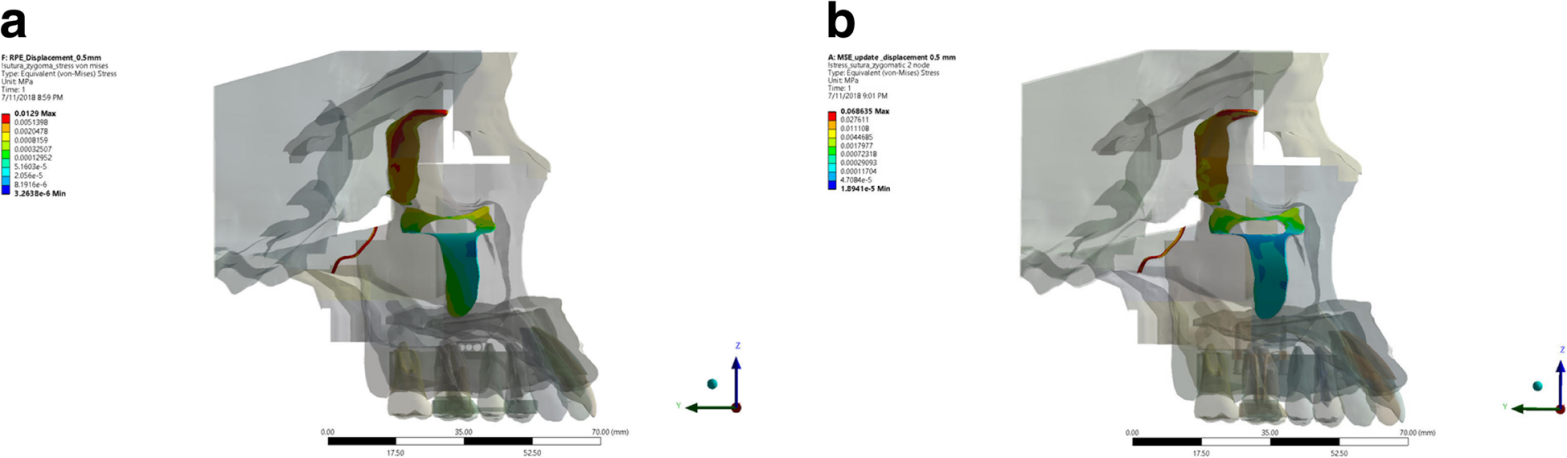
The von Mises stress distribution of the zygomatic suture in the expansion simulation using (**a**) RME, and (**b**) MSE

Expansion simulation in both RME and MSE groups showed similar stress distribution patterns with the highest stress concentration observed at the zygomaticotemporal sutures (Fig. 4).

**Fig. 5:**
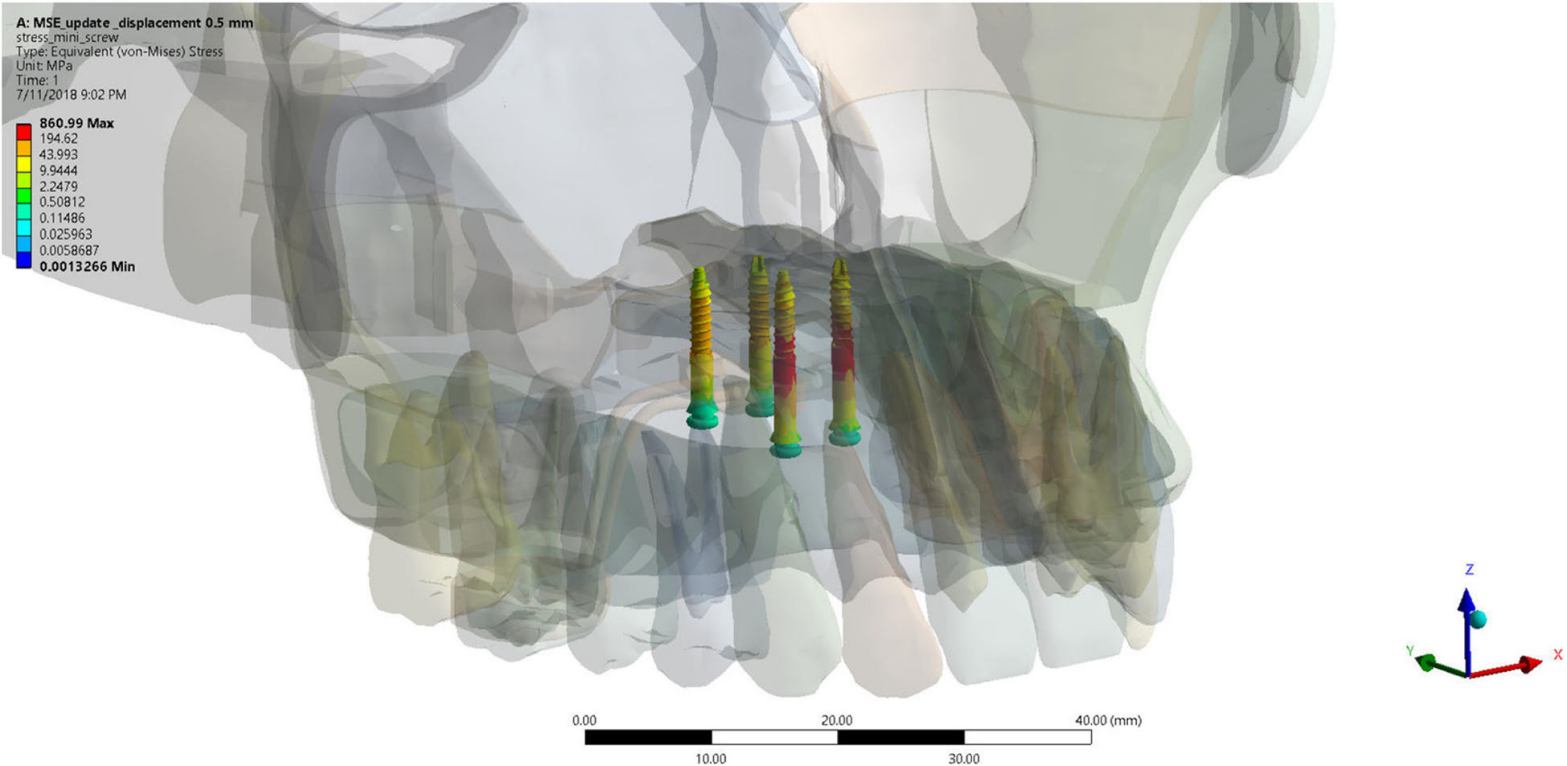
The stress distribution of the miniscrew in the expansion simulation using MSE

On visual observation, the color map of maxillary expansion simulation on miniscrews in MSE group displayed a stress concentration localized at the anterior miniscrews. MaxPS distribution showed left anterior miniscrews as the area that receive the highest tension, while MinPS distribution showed right anterior miniscrews as the area that receive highest compression. The statistical analysis using one-way ANOVA showed a significance value of 0.00 (*p* < 0.05) on every stress tested (Von Ms, MaxPS, MinPS). Therefore, it can be concluded that there was a statistically significant difference of every stress on different displacement tested (Fig. 5).

**Fig. 6:**
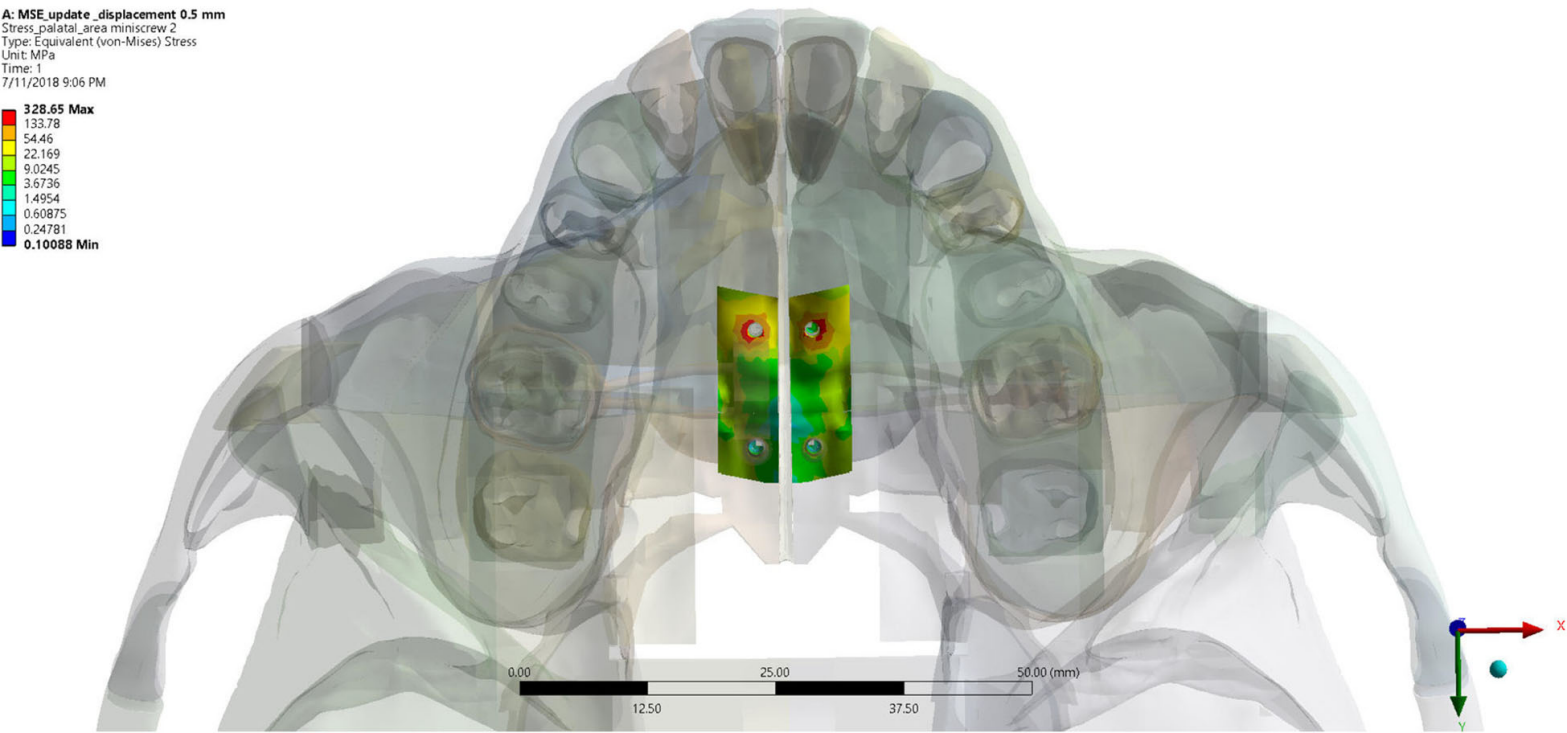
The stress distribution of the palatal bone around miniscrew insertion site in the expansion simulation using MSE

Maxillary expansion simulation using MSE showed stress concentration localized at the palatal area of anterior miniscrews. The statistical analysis using one-way ANOVA showed a significance value of 0.00 (*p* < 0.05) on every stress tested (Von Ms, MaxPS, MinPS). Therefore, it can be concluded that there was statistically significant differences of every stress on different displacement tested (Fig. 6).

## Discussion

There are three types of stress simulate in this study. Maximum principal stress (MaxPS) shows the area undergoing the highest tension, while minimum principal stress (MinPS) shows the area undergoing the highest compression. Von Mises stress on the other hand, shows the area with the highest equivalent stress. MaxPS and MinPS shows tension and compression areas, which are important as they are related to the resorption and apposition process of bone remodeling [19]. Von Mises stress is a value used to determine whether a given material will yield or fracture, which is mostly used for ductile materials [21]. According to Moga (2013), stress distribution and its intensity are important factors in predicting the possibility of root resorption [22].

**Fig. 7:**
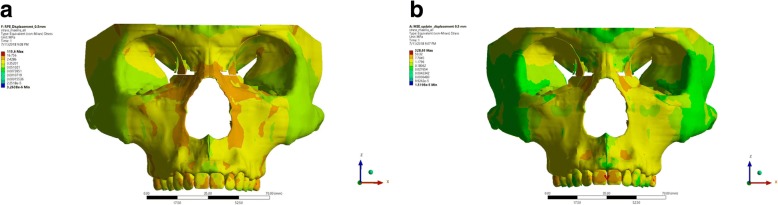
Frontal aspect of stress distribution as the resut of expansion of RME (**a**) and MSE (**b**)

In this research, it was observed that from the frontal aspect, the stress distribution of expansion using RME was concentrated around nasal and infraorbital area (Fig. 7). This was expected, as Garrett et al. (2008) and Mosleh et al. (2014) stated that expansion using RME caused an opening of the maxillary bone with frontonasal suture and midpalatine suture served as its center of rotation [2, 3]. The frontal view of maxillary expansion simulation using MSE displayed equal stress distribution on the maxillary and frontonasal area. Concentration of stress were also observed on the central incisive area, which might be caused by the opening of palatine suture.

**Fig. 8:**
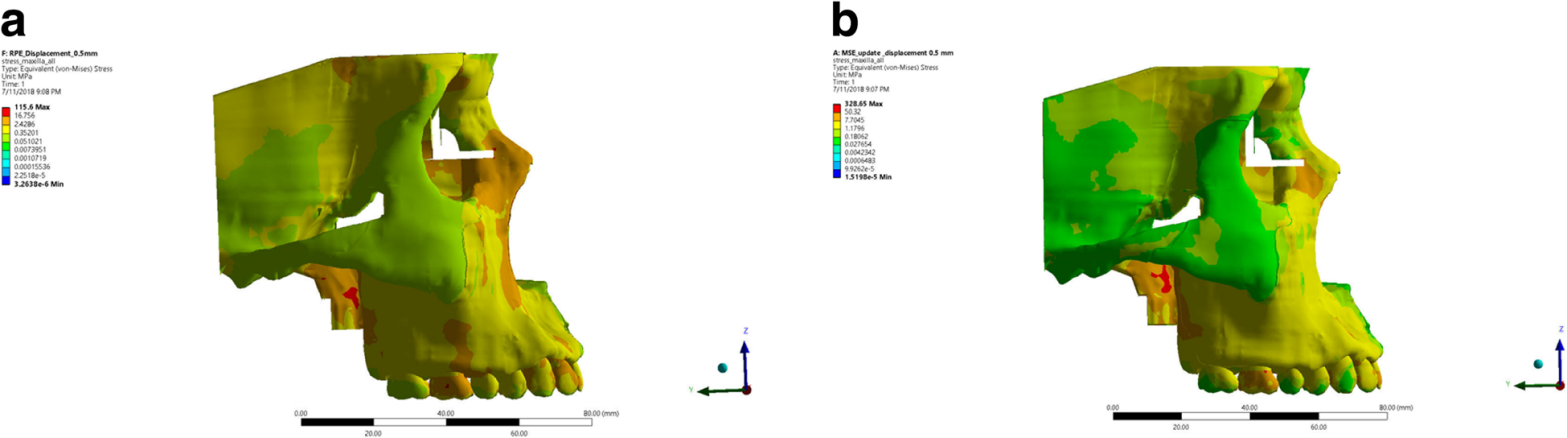
Lateral aspect of stress distribution as the resut of expansion of RME (**a**) and MSE (**b**)

From the lateral view, the stress distribution pattern in the RME group were observed on the temporal bone and maxillary bone, whereas a localized stress concentration were observed in the nasal area, orbital, pterygomaxillary suture, lateral incisive, upper first molar, and buccal cortical bone of upper first molar (Fig. 8). Stress concentration on the buccal cortical bone of the upper first molar was consistent to the study of Lagravere et al. (2010) which stated that expansion with RME will cause larger buccal cortical bone expansion than suture expansion which will manifest on bending of the alveolar bone [11]. Maxillary expansion simulation using RME displays equal stress distribution on the maxillary and frontonasal area, while stress concentration were observed on the buccal bone of the upper first molar and pterygomaxillary suture. On the lateral aspect, no area of stress concentration were observed on buccal cortical bone area; hence, buccal bone bending did not occur as those found on the group using RME.

**Fig. 9:**
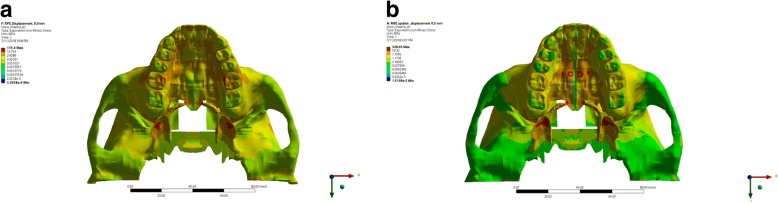
Occlusal aspect of stress distribution as the result of expansion of RME (**a**) and MSE (**b**)

In this research, it was observed that from the occlusal aspect, the stress distribution of expansion using RME was concentrated around the whole maxillary bones, temporal bone, palatal alveolar bone of incisive area to posterior teeth region, pterygoid plate, pterygopalatine suture, and the most significant stress concentration were found on the palatal bone of the upper first molar (Fig. 9). This result was in agreement with the study conducted by Lee et al. [16] which stated that the expansion force is located on the palatal aspect of upper first molar; thus, stress concentration will be found on the junction between the palatine bone and pterygoid plate. Furthermore, from the occlusal aspect, we could observe that the opening of the palatine sutures are only seen on the anterior region, which was consistent with the study conducted by Akkaya et al. (1998) and Wertz (1970) that transverse expansion with RME caused a more significant opening at the anterior region [13, 23–25]. Garrett et al. claimed in his study that suture expansion with RME exhibit a wedge-shaped pattern with wider maxillary anterior [3].

The view from occlusal aspect displayed stress distribution on palatal area, pterygoid plate, and centered stress concentration on miniscrews and pterygoid plate areas. Stress were concentrated around the miniscrews, as the miniscrews served as absolute anchorage on the expansion. The use of miniscrews will transmit direct expansion force on palatal bone thus producing skeletal movement [2]. The view of occlusal aspect displayed a parallel palatine suture opening resembling a straight line from posterior to anterior. This was consistent with study done by Lin which stated that MSE caused a more parallel opening of the sutures when compared to opening produced by RME [15] (Fig. 9).

Overall result of statistical analysis on RME and MSE group showed different stress distribution that were statistically significant on the ROI of the upper first molar, palatal alveolar bone of upper first molar, palatine suture, and zygomatic suture with the exception of MaxPs stress distribution in zygomatic suture on 1.5-mm displacement, and MaxPs stress distribution in palatine suture on 1.5-mm displacement. Visually, no significant difference was depicted in the stress distribution pattern on the color maps of zygomatic suture of expansion using RME and MSE. This was expected, as Angelieri et al. and Gautam et al. stated that palatal suture rupture on an adult would affect circummaxillary suture [26, 27].

Stress distribution on ROI upper first molar as the result of expansion simulation with RME displayed stress concentration on palatal of the upper first molar on both enamel and dentin. This result further verified that molar teeth as the anchorage unit of RME would receive a more localized stress on the palatal area of molar teeth. Moreover, dental tipping might occur as the side effect of expansion using RME. This was consistent with the study of Garett et al. and Sun et al. who stated that expansion using RME will cause skeletal expansion, bending of the alveolar bone, and tipping of the tooth [3, 4].

Stress distribution on zygomatic suture as the result of expansion simulation with RME and MSE showed stress distribution pattern on zygomaticotemporal suture and zygomaticofrontal. Both groups displayed localized stress concentration at zygomaticotemporal suture. However, stress on zygomaticosphenoid suture were observed in the group using RME which were not found in the group using MSE. This was consistent with some research conducted by Zimiring and Isaacson, Chaconas and Caputo, and Lee et al. (2014) that stated the expansion force did not affect the integrity of palatine suture alone, but the whole craniofacial complex that was surrounded by sphenoid and zygomatic bone [13, 28–31].

In this research, on palatine suture, ROI displayed a different pattern of stress distribution between the RME and MSE group. In the RME group, stress was prevalently distributed on nearly the entire palatine suture with the most significant stress concentration found on inferior cortex of palatine suture. Prevalent stress distribution of MSE was found on superior and inferior cortical palatine bone. Numerically, the amount of stress as a result of expansion simulation using MSE was greater than using RME on palatine suture ROI. Moreover, the larger the displacement of the jackscrew, the greater stress observed.

**Fig. 10:**
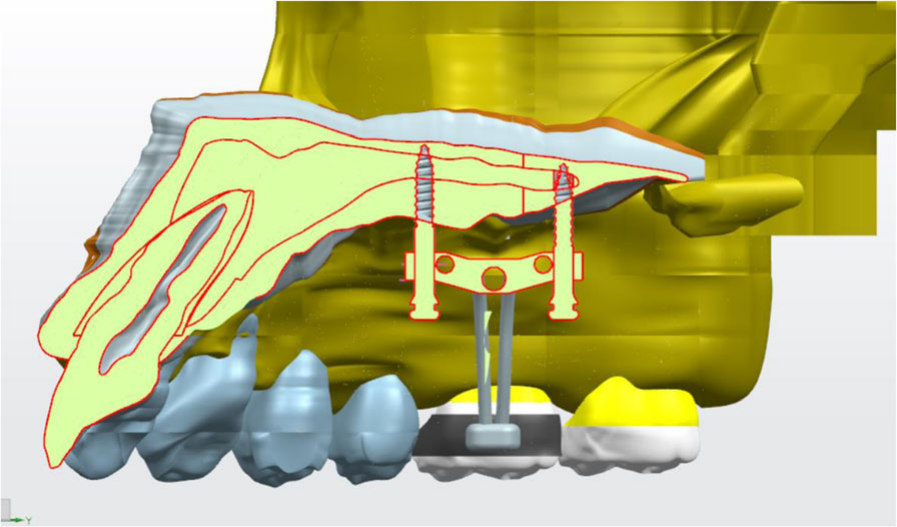
Cortical bone thickness dissimilarity on palatum

From the MSE expansion, simulation showed a parallel palatal suture opening, which is attributed to the usage of 11 mm in length miniscrew that causes a bi-cortical engagement on the palatal bone and nasal base. Stress concentration was centered around two of the anterior miniscrews, which might be caused by the difference in the thickness of the cortical bone resulting in different engagement of anterior and posterior miniscrew. This result was in agreement with Lee et al. which stated that the posterior cortical bone is thinner than the anterior cortical bone [23]. Nevertheless, miniscrews as the absolute anchorage device has been proven to create a more parallel opening of the palatine suture. However, miniscrews remain a tool for absolute anchorage capable for skeletal movement and proven to be able parallel split palatine suture (Fig. 10).

In this research, a detailed model of the craniomaxillary structures was created following the standard template library of the CBCT scan of a dried human skull with minimum simplification. This study showed the potential benefit for clinician to use MSE in comparison to RME such as the possible alleviation of stress distribution on the buccal bone (as seen in Fig. 8), parallel split of the palatine suture (as seen on Fig. 9), and minimized tipping of the teeth (as seen in Fig. 1). Therefore, the use of MSE might be considered beneficial for maxillary expansion on non-growing patients with possible more skeletal effects.

## Conclusions

There seemed to be significant differences of stress distribution for the RME group compared to the MSE group. The differences of stress distribution both visually and statistically were found on the upper first molar region of interest (ROI), palatal alveolar bone of first molar teeth, palatinal suture, and zygomatic sutures. From this, research showed the potential benefit for a clinician to use MSE in comparison to RME such as the possible alleviation of stress distribution on the buccal bone, parallel split of the palatine suture, and minimized tipping of the teeth. Thus, MSE tools might be recommended for non-growing patients with possible more skeletal effects.
